# Ferroptosis, a new target for treatment of renal injury and fibrosis in a 5/6 nephrectomy-induced CKD rat model

**DOI:** 10.1038/s41420-022-00931-8

**Published:** 2022-03-22

**Authors:** Jingyu Wang, Yaqing Wang, Yi Liu, Xintian Cai, Xin Huang, Wenjing Fu, Lei Wang, Lihua Qiu, Junying Li, Li Sun

**Affiliations:** 1 Department of Nephrology, The First Affiliated Hospital of China Medical University, Shenyang, 110000 Liaoning Province People’s Republic of China; 2 Department of Anesthesiology, Affiliated Hospital of North Sichuan Medical College, Nanchong, 637000 Sichuan Province People’s Republic of China; 3 Department of Graduate School, Xinjiang Medical University, Urumqi, 830054 Xinjiang Province People’s Republic of China; 4 Department of Nephrology, The Fourth Affiliated Hospital of China Medical University, Shenyang, 110000 Liaoning Province People’s Republic of China; 5 Department of epigenetics, China Medical University, Shenyang, 110000 Liaoning Province People’s Republic of China

**Keywords:** Chronic kidney disease, Experimental models of disease

## Abstract

Ferroptosis is a non-traditional form of regulated cell death, characterized by iron overload and lipid peroxidation. Exploration of ferroptosis in chronic kidney disease (CKD) has been extremely limited to date. In this study, we established a rat model of CKD by 5/6 nephrectomy, treated CKD rats with the ferroptosis inducer, cisplatin (CDDP), and the ferroptosis inhibitor, deferoxamine mesylate (DFO), and observed the resulting pathologic changes (injury markers and fibrosis) and ferroptotic biochemical indices. Kidney iron deposition, lipid peroxidation, mitochondrial defects, ferroptosis marker induction, and TUNEL staining positivity were detected in CKD group rats. Further, treatment with CDDP or DFO influenced renal injury and fibrosis by affecting ferroptosis, rather than apoptosis, and ferroptosis occurs in the remnant kidney due to disordered iron metabolism. In conclusion, our study shows for the first time that 5/6 nephrectomy induces ferroptosis in the remnant kidney and clarifies the underlying pathogenesis. Moreover, we demonstrate that ferroptosis is involved in CKD progression and represents a therapeutic target in chronic kidney injury and renal fibrosis.

## Introduction

The physiological functions of iron include participation in the mitochondrial respiratory chain and hemoglobin synthesis [1], and overload and deficiency of iron are detrimental to metabolic homeostasis. Excess iron triggers free radical production, which damages macromolecules [2], and can also lead to organelle stress [3, 4], disrupting cellular structural integrity and tissue homeostasis. Patients with chronic kidney disease (CKD) develop renal anemia and reduced erythropoietin secretion. Decreased appetite, and frequent dialysis and punctures render correction of iron deficiency challenging. Combating renal anemia positively can improve the life-quality and survival of CKD patients; hence, alleviation of circulating iron deficiency has long been a hot research topic.

Although renal dysfunction contributes to decreased iron bioavailability, kidney cells are susceptible to iron overload [5–7], and tissue iron deposition causes oxidative damage and pathological responses, including fibrosis and inflammation [8–10]. Therefore, mitigating tissue iron deposition appears more effective in alleviating oxidative insults than ameliorating circulating iron deficiency.

Treatment of tumor cells with Erastin induces tumor cell death by a process distinct from apoptosis [11]. Erastin activity is dependent on mitochondrial voltage-dependent anion channel 3 (VDAC3) and involves redox disruption [12]. VDAC3 also contributes to Ras-selective lethal (RSL)-mediated cell death; increased intracellular iron content is detected after RSL-mediated oncogenic transformation, and iron chelators are effective in rescuing the cell death [13]. This type of cell death is referred to as ferroptosis and is associated with iron accumulation and lipid peroxidation [14]. Erastin, RSL3, and cisplatin (CDDP) are selective or non-selective inducers of ferroptosis [15–17], while iron chelators, including deferoxamine mesylate (DFO), are ferroptosis inhibitors [15]. Ferrous iron, total reactive oxygen species (T-ROS), lipid ROS (L-ROS), malondialdehyde (MDA), 4-hydroxynonenal (4-HNE), reduced glutathione (GSH), glutathione peroxidase 4 (GPX4), solute carrier family 7 member 11 (SLC7A11), and acyl-CoA synthetase long-chain family member 4 (ACSL4) are involved in, or inhibit, ferroptosis. Mitochondrial phenotype defects, including mitochondrial volume (MV) reduction, mitochondrial outer membrane (MOM) rupture, and decreased or absent mitochondrial cristae (MC), are direct pathological evidence of ferroptosis [18].

Ferroptosis participates in the folic acid-induced acute kidney injury (AKI) mouse model [19], and is implicated in AKI mediated by pathological factors, including rhabdomyolysis and ischemia-reperfusion injury [20]. AKI and CKD are interrelated; AKI promotes CKD development and CKD increases susceptibility to AKI [21]. Therefore, we hypothesize that ferroptosis is also present in CKD. The relationship between CKD and ferroptosis was discussed in our previous study [22]; however, exploration of ferroptosis in CKD remains limited.

The 5/6 nephrectomy operation induces CKD, closely mimicking the hyperperfusion, hyperfiltration, and hypertensive status of the residual nephron, which develops chronic interstitial fibrotic lesions. Therefore, we used a 5/6 nephrectomy rat model to explore the role of ferroptosis in CKD and the underlying pathological mechanisms.

## Results

### CKD rats exhibit iron accumulation, oxidative stress, and lipid peroxidation, which are altered by CDDP and DFO treatment

**Fig. 1:**
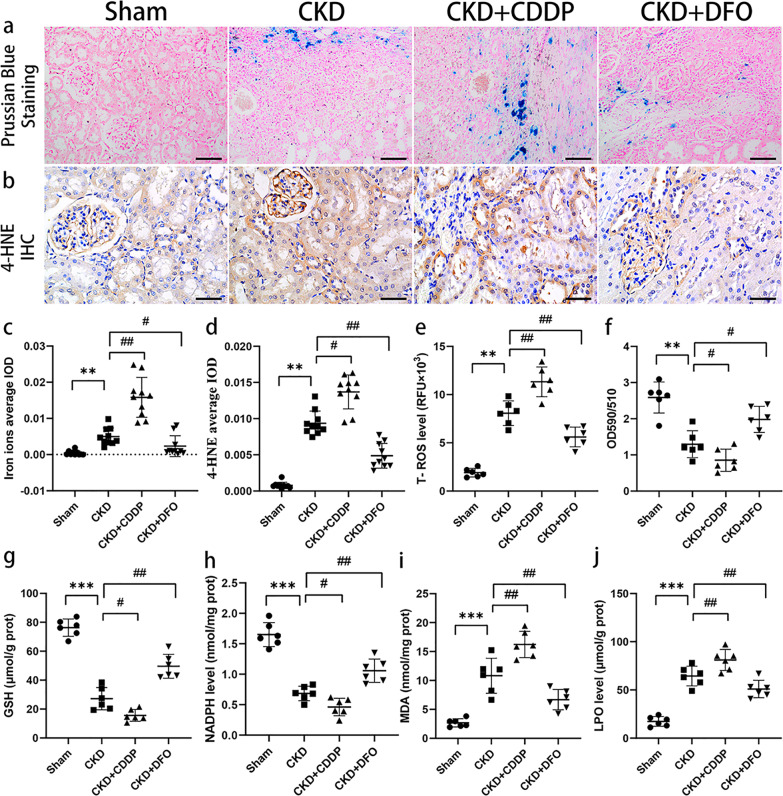
CKD rats exhibit iron accumulation, oxidative stress, and lipid peroxidation, which are altered by CDDP and DFO treatment. CKD rats developed iron accumulation (**a**, **c**), oxidative stress (**e**, **g**, and **h**), and lipid peroxidation (**b**, **d**, **f**, **i**, and **j**), which were aggravated and alleviated by CDDP and DFO, respectively. **P < 0.01, ***P < 0.001 vs. Sham group; ^#^*P* < 0.05, ^##^*P* < 0.01 vs. CKD group. Scale bar in (**a**) = 100 μm. Scale bar in (**b**) = 50 μm.

Spontaneous iron deposition was observed in CKD rats and was enhanced or attenuated by CDDP or DFO treatment, respectively (Fig. 1a, c). Treatment with CDDP and DFO also resulted in corresponding changes in T-ROS, L-ROS, 4-HNE, MDA, and LPO (Fig. 1b, d–f, i, and j).

GSH and NADPH exhibit anti-oxidative stress and anti-ferroptotic effects due to their involvement in GPX4 and mevalonate biosynthesis, respectively. GSH and NADPH levels were lower in CKD than Sham control group rats, and the reductions were exacerbated by CDDP and reversed by DFO (Fig. 1g, h).

These data indicate that CKD rats develop renal iron accumulation, oxidative stress, and lipid peroxidation, which are characteristic of ferroptosis.

### Typical features of ferroptosis present in CKD rats

**Fig. 2:**
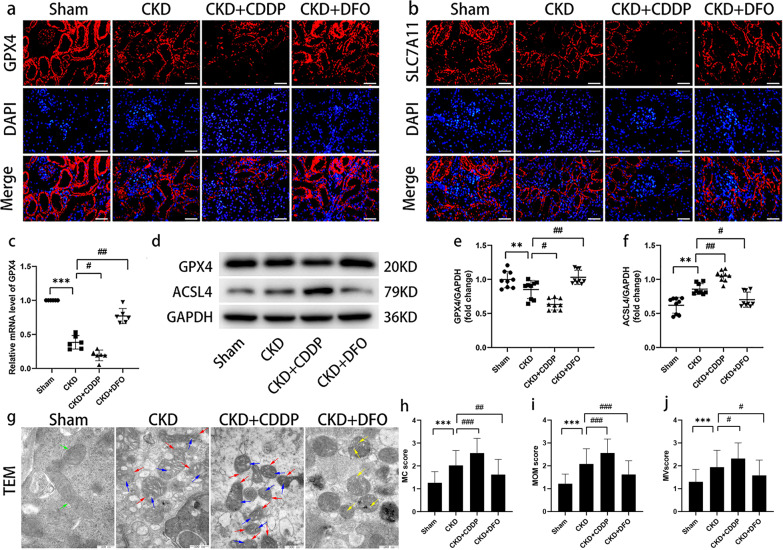
Typical features of ferroptosis present in CKD rats. Biochemical trends (**a**–**f**) and pathological evidence (**g**–**j**) of ferroptosis in CKD rats. Green arrow, normal mitochondria; red arrow, MOM rupture; blue arrow, reduction or loss of MC; yellow arrow, MC swelling. **P < 0.01, ***P < 0.001 vs. Sham group; ^#^*P* < 0.05, ^##^*P* < 0.01, ^###^*P* < 0.001 vs. CKD group. Scale bar in (**a**) = 50 μm. Scale bar in (**f**) = 500 μm.

Given the close association between ferroptosis and oxidative stress, we sought further evidence of ferroptosis in CKD rats. IF analysis demonstrated that GPX4 and SLC7A11 expression was concentrated in tubular cells and decreased sequentially from the Sham, to the CKD + DFO, CKD, and CKD + CDDP groups (Fig. 2a, b). The cystine/glutamate antiporter (system Xc-), comprising SLC7A11 and SLC3A2 subunits, requires NADPH to capture extracellular cystine (Cys) for conversion to cytoplasmic cysteine for GSH synthesis; therefore, the reduced GSH synthesis and diminished GPX4 activity in the CKD group may be attributable to NADPH and SLC7A11 deficiency.

WB and RT-qPCR assays of GPX4 generated results consistent with those of IF (Fig. 2c–e), whereas the expression trend of ACSL4 was opposite to that of GPX4, with lowest expression in the Sham group and highest in the CKD + CDDP group (Fig. 2d, f).

Evaluation of mitochondrial morphology indicated significant differences among groups (Fig. 2g–j). MOM were intact and MC were clear in the Sham group, while the mitochondria became smaller, MOM ruptured, and the cristae were reduced or absent in the CKD group. In the CKD + CDDP group, mitochondria were more crumpled and the MOM rupture and MC disappearance more pronounced, while mitochondrial defects were reversed after DFO treatment. These data suggest that specific pathological manifestations of ferroptosis occur in CKD rats.

### CDDP and DFO affect 5/6 nephrectomy-induced ferroptosis, independent of apoptosis

**Fig. 3:**
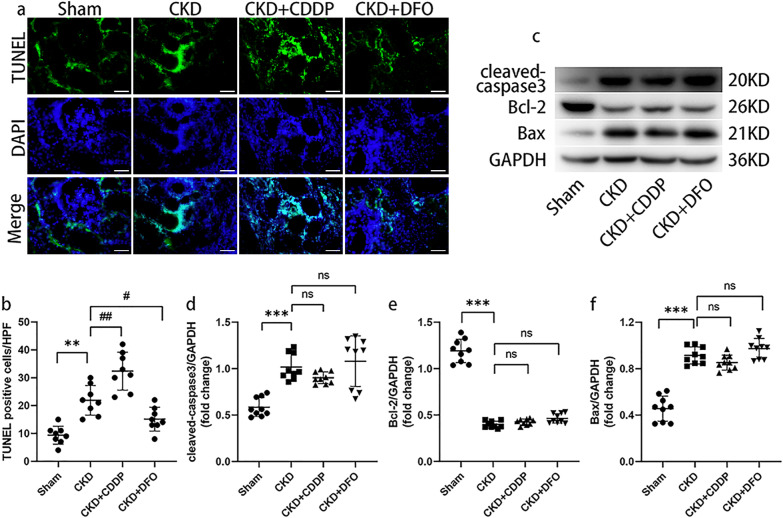
CDDP and DFO affect 5/6 nephrectomy-induced ferroptosis, independent of apoptosis. CDDP and DFO affect 5/6 nephrectomy-induced ferroptotic cell death (**a**, **b**), rather than apoptosis (**c**–**f**). **P < 0.01, ***P < 0.001 vs. Sham group; ^#^*P* < 0.05, ^##^*P* < 0.01 vs. CKD group. ns: *P* > 0.05, no significance. Scale bar = 50 μm.

Ferroptosis can cause DNA double-strand breaks, which appear positive on TUNEL staining [23, 24]. There were significantly more TUNEL-positive cells in the CKD group than the Sham group, while CDDP and DFO increased and decreased the number of TUNEL-positive cells, respectively (Fig. 3a, b).

Pro-apoptotic proteins including cleaved-caspase3 and Bax were significantly up-regulated in the CKD group compared to the Sham group, as opposed to the anti-apoptotic protein Bcl-2; however, CDDP and DFO intervention failed to change the expression levels of apoptotic proteins (Fig. 3c–f).

These data suggest that ferroptosis and apoptosis both occurred in the remnant kidney, and that CDDP and DFO intervention had no effect on apoptosis.

### Ferroptosis occurs in the remnant kidney due to disordered iron metabolism, which are altered by CDDP and DFO treatment

**Fig. 4:**
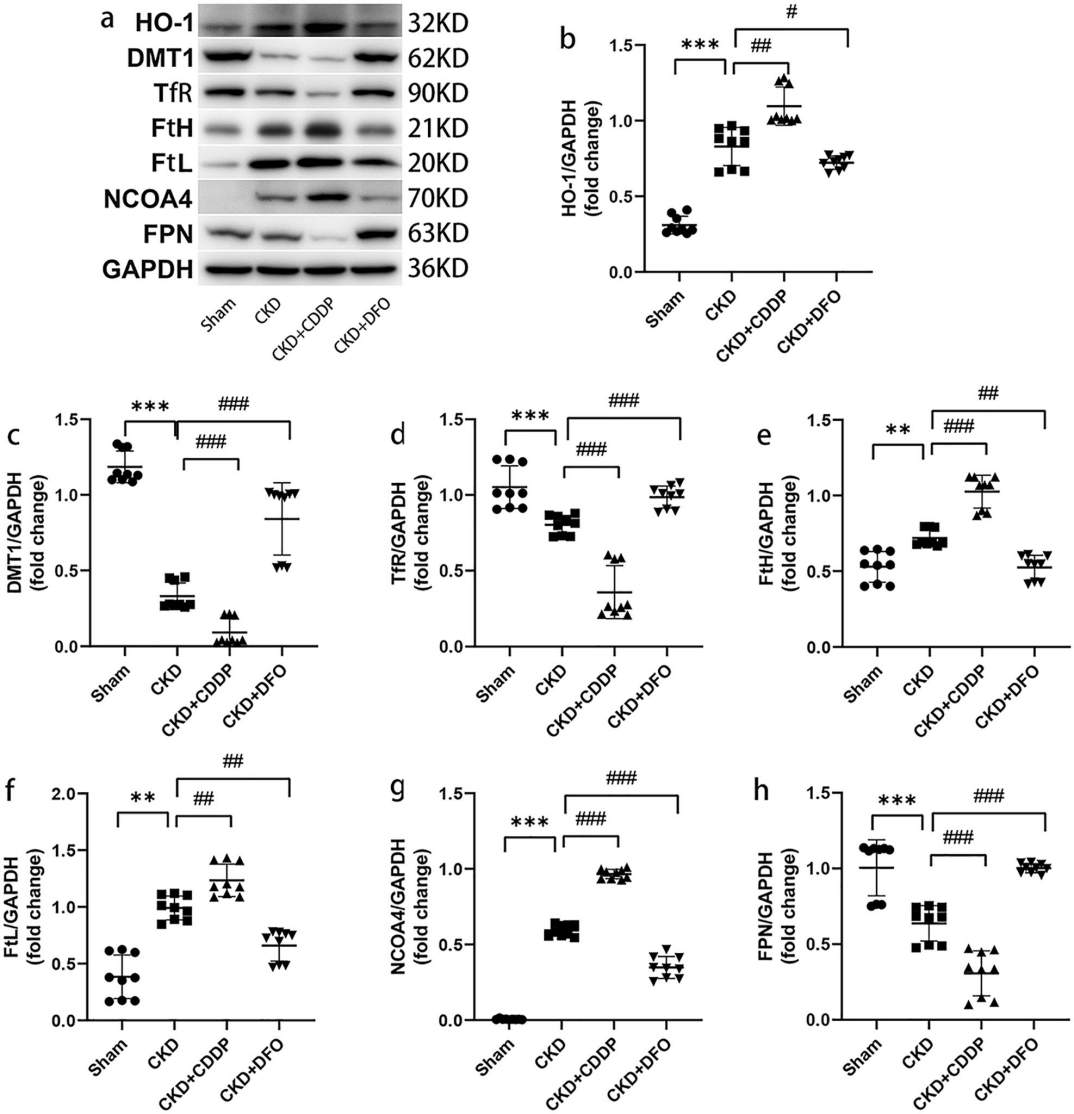
Ferroptosis occurs in the remnant kidney due to disordered iron metabolism, which are altered by CDDP and DFO treatment. Disordered iron metabolism (**a**–**h**) in the remnant kidney is responsible for ferroptosis, which are aggravated and alleviated by CDDP and DFO, respectively. **P < 0.01, ***P < 0.001 vs. Sham group; ^#^*P* < 0.05, ^##^*P* < 0.01, ^###^*P* < 0.001 vs. CKD group.

We performed a relatively comprehensive assay of iron metabolism proteins in the kidneys to elucidate the mechanisms of ferroptosis pathogenesis. Up-regulation of HO-1 likely mediated increased release of divalent iron from heme in the CKD group, whereas up-regulation of FtH and FtL would facilitate nucleation and mineralization of excess divalent iron, which would be sequestrated by FtH (Fig. 4a, b, e, and f).

We also measured expression levels of DMT1 and TfR. Surprisingly, levels of DMT1 and TfR decreased following CKD development, were reduced by CDDP intervention, and were significantly restored after treatment with DFO (Fig. 4a, c, and d), suggesting that, DFO, unlike CDDP, can facilitate restoration of iron homeostasis.

Levels of FPN, the only iron export protein in mammals, were down-regulated in the CKD group and the down-regulation was remarkably aggravated and reversed by CDDP and DFO treatment, respectively (Fig. 4a, h), indicating that iron deposition in the remnant kidney of CKD rats may be attributable to FPN down-regulation, and that DFO can ease tissue iron overload by up-regulating FPN.

NCOA4 is an important mediator of ferritinophagy, and WB analysis of NCOA4 indicated that ferritinophagy occurs in CKD and can be enhanced or down-regulated by CDDP or DFO treatment, respectively (Fig. 4a, g). Hence, ferritinophagy may contribute to ferroptosis in CKD.

These data suggest that disordered iron metabolism occurred in the remnant kidney, and that NCOA4 up-regulation and FPN down-regulation may be important contributors to tissue iron accumulation and ferroptosis.

### Ferroptosis is a potential therapeutic target in chronic kidney injury

**Fig. 5:**
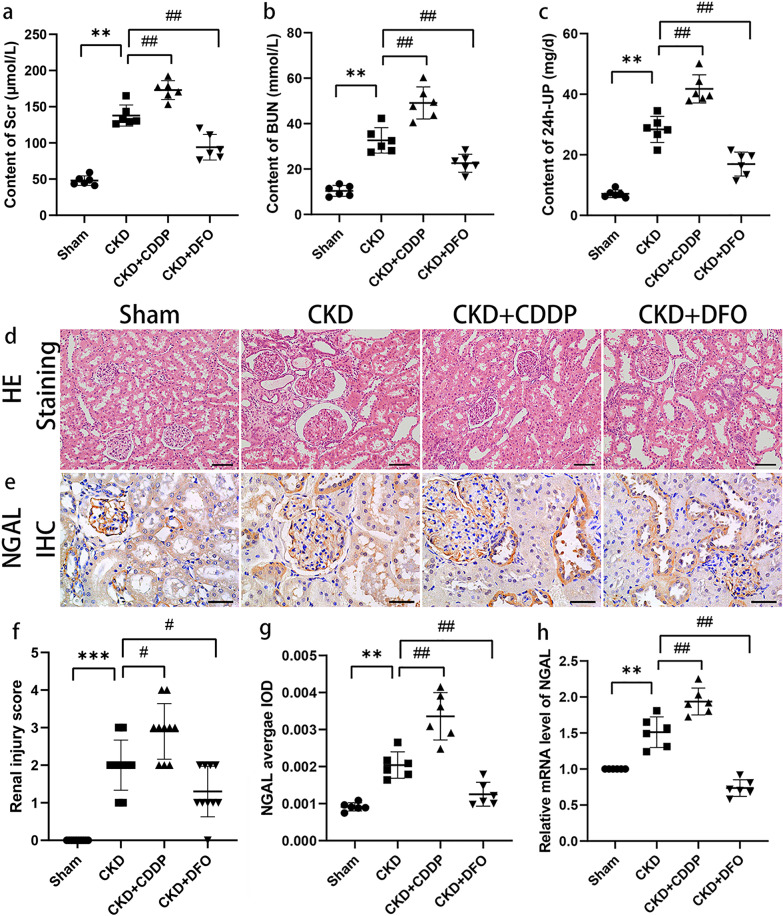
Ferroptosis is a potential therapeutic target in chronic kidney injury. CDDP and DFO aggravate and reverse 5/6 nephrectomy-induced chronic kidney injury, respectively (**a**–**h**). **P < 0.01, ***P < 0.001 vs. Sham group; ^#^*P* < 0.05, ^##^*P* < 0.01 vs. CKD group. Scale bar = 50 μm.

Levels of SCr, BUN, and 24-UP were significantly higher in the CKD group than the Sham group, and treatment with CDDP exacerbated the decline in renal function, while DFO treatment ameliorated it (Fig. 5a–c).

Histopathological analysis supported these effects on renal function (Fig. 5d, f); few pathological injuries were detected in the Sham group, while glomerular injury and tubular epithelial cell expansion or atrophy were observed in CKD rats, and these effects were aggravated by CDDP and partially mitigated by DFO.

Expression levels of the marker of kidney injury, NGAL, were low in renal tubular epithelial cells under physiological conditions and significantly elevated after development of renal injury (Fig. 5e, g, and h). NGAL mediates intracellular iron ion transport [25], consistent with the observed iron accumulation in CKD rat residual kidney.

Since intervention with CDDP or DFO did not influence apoptosis, together with the data presented in Figs. 1 and 2, our findings suggest that CDDP and DFO influence kidney injury progression by targeting ferroptosis, highlighting ferroptosis as a potential target in treatment of chronic kidney injury.

### Ferroptosis is a potential therapeutic target in renal fibrosis

**Fig. 6:**
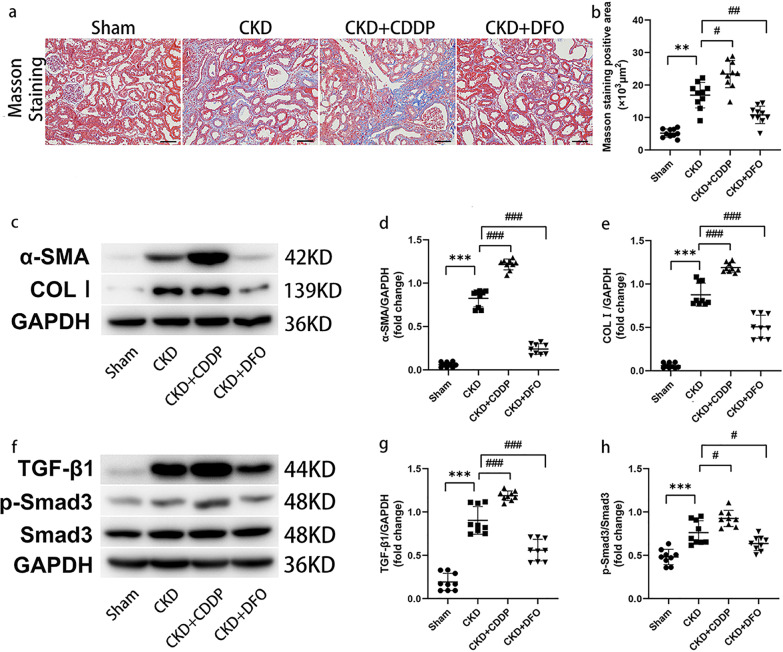
Ferroptosis is a potential therapeutic target in renal fibrosis. CDDP and DFO affect fibrosis progression (**a**, **b**) and ECM deposition (**c**–**e**) in CKD rats by regulating the TGF-β1/Smad3 signaling pathway (**f**–**h**). **P < 0.01, ***P < 0.001 vs. Sham group; ^#^*P* < 0.05, ^##^*P* < 0.01, ^###^*P* < 0.001 vs. CKD group. Scale bar = 50 μm.

Masson staining revealed only trace amounts of blue-stained collagen fibers in the Sham group, while in the CKD group, renal fibrosis was manifested as a mass of blue-stained collagen fibers in the tubular interstitium (Fig. 6a, b). Renal interstitial fibrosis was aggravated by CDDP treatment, while DFO had anti-fibrotic effects. WB demonstrated that CDDP exacerbated and DFO inhibited deposition of the ECM proteins, α-SMA and COL I (Fig. 6c–e).

TGF-β1 induction contributes to fibrotic event progression and TGF-β1 is a critical pro-fibrotic factor. TGF-β1 and p-Smad3 levels were highest in the CKD + CDDP group, while DFO reduced TGF-β1/Smad3 induction in CKD (Fig. 6f–h).

Since CDDP and DFO interventions did not affect apoptosis, these findings suggest that ferroptosis is a potential target for treatments aimed at delaying renal fibrosis progression, and that the TGF-β1/Smad3 signaling pathway is important in this context.

## Discussion

With the substantial improvements in living standards brought about by rapid global economic development, the prevalence rates of numerous metabolic diseases, such as obesity, diabetes, hypertension, hyperlipidemia, and hyperuricemia have increased, leading to a burden on the kidneys and growing CKD incidence and prevalence [26, 27]; CKD is expected to rise to be among the top five causes of mortality by 2040 [28]. The contrast between the growing number of patients with CKD and the limited treatment options is becoming increasingly stark, and research into the causes of kidney cell death is imperative.

Apoptosis is a common form of renal cell death, the mitochondrial pathway is essential in apoptotic signaling, and the Bcl-2 protein family are major regulators of this pathway [29]. Bcl-2 can influence MOM permeability and cytochrome c release, protecting cells from apoptosis by inhibiting caspase-3 activation and the corresponding downstream cascade [30]. In our study, cleaved-caspase3 and Bax up-regulation, and Bcl-2 down-regulation were observed in the CKD group, suggesting that both apoptosis and ferroptosis contribute to chronic kidney injury progression and are responsible for pathological injury and interstitial fibrosis; however, neither CDDP nor DFO treatments altered apoptotic protein expression, although these interventions have previously shown pro- and anti-apoptotic effects, respectively [31, 32]; differences in dosing frequency, dosage, and/or experimental subjects most likely account for this discrepancy.

**Fig. 7:**
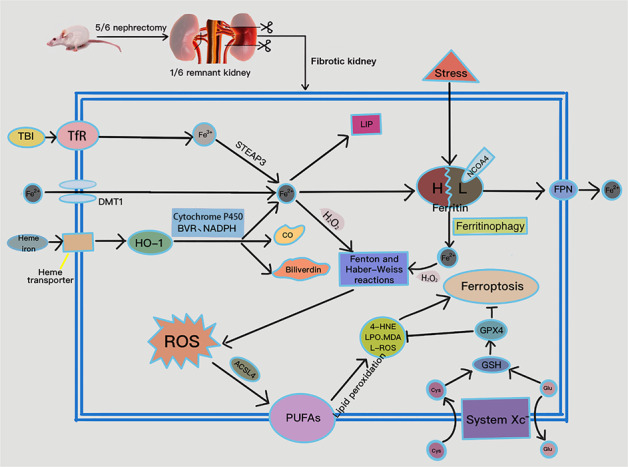
Disordered iron metabolism and ferroptosis occurred in 5/6 nephrectomy-induced CKD rats. DMT1 and TfR expression were down-regulated, while HO-1 was up-regulated; the latter catabolizes heme iron to ferrous iron, CO, and biliverdin, with the assistance of cytochrome P450, biliverdin reductase (BVR), and NADPH. TfR mediates the import of transferrin-bound iron (TBI) into the cell and reduces ferric iron to ferrous iron via six-transmembrane epithelial antigen of prostate 3 (STEAP3). Regarding iron export, FPN down-regulation blocks iron efflux, leading to elevated intracellular iron concentration. A small amount of intracellular ferrous iron is stored in the labile iron pool (LIP) to maintain physiological metabolism, while the remainder is endogenously chelated by ferritin. Excess intracellular catalytic iron reacts with hydrogen peroxide to initiate the Fenton and Haber-Weiss reactions, leading to a further increase in ROS and attacks on polyunsaturated fatty acids (PUFAs) in the cytomembrane, initiating lipid peroxidation and ferroptosis. In addition, down-regulation of SLC7A11 results in reduced synthesis of GSH from Cys, decreased GPX4 activity, and weakened anti-ferroptotic responses. CO carbon monoxide, Glu glutamate.

Iron metabolism in CKD is extremely complex. In our study, expression of DMT1 and TfR decreased with CKD progression, and FtH and FtL increased with CKD progression. Theoretically, the above changes are not conducive to ferroptosis. Reportedly, DMTI was up-regulated in the kidneys of unilateral ureteral obstruction-induced CKD mice [33, 34]. While in human kidney biopsy samples, DMT1 expression was not consistent in different types of CKD [35]. The above suggests that different pathological types and modeling approaches may be important reasons for the differences in expression of DMT1. Expression of TfR was low in the CKD group, indicating that the kidney may be under an elevated level of negative feedback control during local iron overload. Increased ferritin expression in CKD may be related to the activation of the NF-κB pathway and the degradation of iron-responsive proteins by inflammatory cytokines [36–39]. Our study demonstrates that ferroptosis occurs, despite high expression of some negative regulators of this process, due to disruption of iron uptake, storage, and excretion homeostasis (Fig. 7). Hence, more comprehensive analysis of iron metabolism-related proteins in CKD pathology is required.

**Fig. 8:**
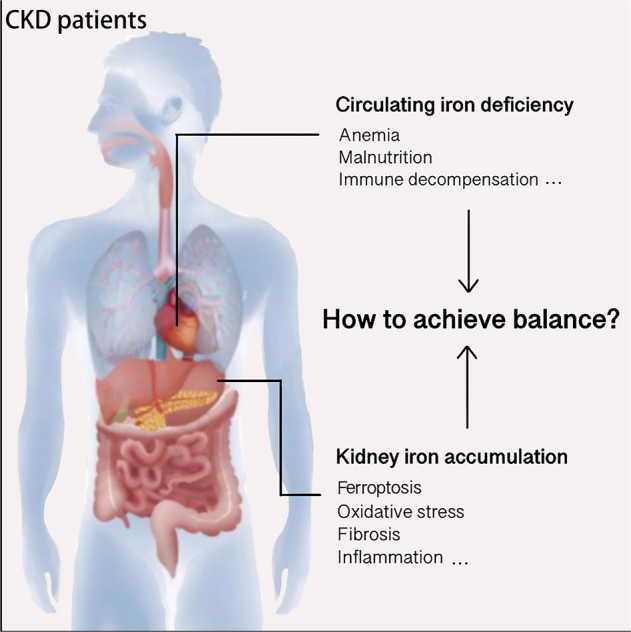
Opposite iron status in the kidney and blood circulation of patients with CKD.

In patients with CKD, renal iron accumulation and circulating iron deficiency contradict one another, and these opposing iron saturation characteristics have different consequences (Fig. 8). Given the uniqueness of FPN at iron export ports, this molecule is crucial to the exchange of iron between tissue cells and plasma, and improving low FPN induction in CKD is beneficial [40]. There are well-established detection methods and indices for iron deficiency; however, there remains a lack of validated assessment tools for tissue iron accumulation. Hence, development of specific markers for organ iron accumulation disorders would be of considerable significance.

Interfering with ferroptosis is an emerging therapeutic approach for multiple pathological disorders. Ferroptosis is intertwined with other forms of cell death; therefore, comprehensive evaluation of iron accumulation, oxidative stress, lipid peroxidation, mitochondria-specific defects, cell death/cell viability, and ferroptotic-specific markers could better characterize ferroptosis development, which is of great importance in related diseases. Here, CDDP and DFO affected tissue iron deposition, providing fundamental evidence supporting their effects on ferroptotic cell death. To our knowledge, our data are the first pathological evidence that CDDP exacerbates tissue iron deposition.

Our research has some limitations. First, we did not assess changes in the LIP. Second, the crosstalk among fibrotic signals during renal ferroptosis remains unclear. These limitations remains to be addressed in the future.

In conclusion, we report the first observation of ferroptosis in the 5/6 nephrectomy-induced CKD model, and demonstrate that disordered iron metabolism is an important contributor to this process. We identified the induction of NCOA4 in this context for the first time, suggesting the presence of ferritinophagy in CKD rats. We also show that CDDP and DFO can affect ferroptosis in in vivo experiments and thereby interfere with renal injury and fibrosis progression via regulation of iron metabolism and the TGF-β1/Smad3 pathway. These findings suggest that ferroptosis is a promising target for the treatment of CKD.

## Materials and methods

### Animals and protocols

Male Sprague–Dawley rats (7-week-old; weight, ~220 g) were housed in a climate-controlled room (22 °C ± 1 °C, humidity 45–55%, 12-h day/night cycle), and given free access to water and food. After one week of acclimatization, rats were randomly divided into Sham, CKD, CKD + CDDP, and CKD + DFO groups (*n* = 6 per group). Rats in the CKD + CDDP and CKD + DFO groups were injected intraperitoneally with CDDP (2 mg/kg, batcn number: 0J0216B02, QILU Pharmaceutical Co., Ltd, China) and DFO (300 mg/kg, HY-B0988, MCE, USA), respectively, once per week for 8 weeks; rats in the Sham and CKD groups were injected with an equivalent amount of saline. At the end of experiments, 24-h urine, kidney tissue, and serum were collected for analyses.

### CKD model construction

For 5/6 nephrectomy, rats were anesthetized by intraperitoneal injection of 2% sodium pentobarbital (40–50 mg/kg), and immobilized prone on pre-sterilized workbenches after satisfactory anesthesia. The surgical area was shaved, disinfected with iodophor, and sterile cavity wipes laid. A longitudinal 1.5 cm surgical incision was made next to the spine at the lower left costal margin, and the subcutaneous fascia and muscles sequentially incised to expose the left kidney, which was gently lifted out of the abdominal cavity by holding the perirenal fat with forceps, and the adrenal glands and perirenal membrane carefully peeled off. Approximately two-thirds of the parenchyma at the upper and lower poles of the kidney was removed and the remnant kidney immediately compressed with a gelatin sponge until hemostasis ceased, then returned into the abdominal cavity; only the gerota fascia was stripped in the Sham group. Muscle and skin were sutured layer-by-layer, the surgical incision disinfected, and rats replaced in cages when they awoke. After 1 week, a longitudinal 1.5 cm surgical incision was made next to the spine at the right inferior rib cage of the rat, and the right kidney exposed by isolating the subcutaneous tissue layer-by-layer, as in the previous operation. Following ligation of the renal pedicle with 4–0 silk, renal vessels were cut and the right kidney removed. The abdominal cavity was then closed layer-by-layer, when there was no bleeding, and rats returned to their cages after awakening. In the Sham group, surgical incisions were made without kidney removal. Subcutaneous injection of 0.2 mg/kg buprenorphine was administered to rats every 12 h postoperatively for two days for analgesia. All efforts were made to minimize the suffering of the animals.

### T-ROS and L-ROS assays

For T-ROS measurement, single cell suspensions were generated from renal tissue samples and the cells loaded with the fluorescent ROS probe, DCFH-DA (E004, Nanjing Jiancheng Bioengineering Institute, China), after quantification of protein levels. Cells were cultured (30 min, 37 °C, in darkness) centrifuged (1000 *g*) and supernatants removed. Cell pellets were re-suspended in PBS and T-ROS detected using a Multifunctional enzyme-labeled instrument (excitation, 490 nm; emission, 530 nm; M200PRO, TECAN, Switzerland);.

L-ROS levels were evaluated using an Image-iT™ Lipid Peroxidation Kit (C10445, Invitrogen, USA), based on the manufacturer’s instructions. Results are expressed as the ratio of fluorescence intensity at 590 and 510 nm, following detection using a Multifunctional enzyme-labeling instrument (SynergyH1, Biotek, USA); the 590/510 nm ratio is negatively correlated with lipid peroxidation.

### Analyses of GSH, MDA, nicotinamide adenine dinucleotide phosphate (NADPH), and lipid peroxidation products (LPO)

Renal tissue samples were homogenized and protein levels quantified using a BCA assay kit (PC0020, Solarbio, China). Assay kits (GSH, WLA105, Wanleibio, China; MDA, WLA048, Wanleibio, China; NADPH, A115-1, Nanjing Jiancheng Bioengineering Institute, China; LPO, A106-1-1, Nanjing Jiancheng Bioengineering Institute, China) were used to determine the levels of GSH, NADPH, MDA, and LPO, according to the manufacturer’s protocols.

### Transmission electron microscopy (TEM)

Renal tissues were incubated with 2.5% glutaraldehyde (2 h), post-fixed in 1% osmium acid (1.5 h), dehydrated in ethanol and acetone, and subsequently sectioned and stained with uranium acetate and lead citrate. Representative images showing mitochondrial alterations were captured by TEM (magnification, 40,000×). Fifty mitochondria in renal tubular epithelial cells were randomly selected from ten unduplicated views for morphological evaluation. MV, MOM, and MC were scored from 1 to 3 as follows: 1, normal MV, intact MOM, regular MC; 2, smaller MV, ruptured MOM, decreased MC; 3, significantly smaller MV, pronounced ruptured MOM, absent MC.

### Kidney function assay

Levels of blood urea nitrogen (BUN), serum creatinine (SCr), and 24-h urine protein (24 h-UP) were determined using assay kits (C013-2-1, C011-2-1, C035-2-1, Nanjing Jiancheng Bioengineering Institute, China), according to the manufacturer’s protocols.

### Kidney histopathology analysis

Renal tissue was fixed in 4% paraformaldehyde (24 h), embedded in paraffin, and cut into 4-μm–thick sections, which were deparaffinized by xylol and hydrated in gradient ethyl alcohol. Sections were stained using Hematoxylin-Eosin (HE) and Masson’s Trichrome (Masson) kits (G1120, G1340, Solarbio, China). Following dehydration, clearing, and sealing, representative images were captured by microscopy (DP73, OLYMPUS, Japan; magnification, 200×). Renal injury scores were determined based on previously described indices [41], including inflammatory cell filtration and interstitial fibrosis. Each parameter was scored from 0 to 4 in 10 different randomly selected views (0, impairment < 5%; 1, impairment 5–25%; 2, impairment 26–50%; 3, impairment 51–75%; 4, impairment >75%). Masson staining was analyzed semi-quantitatively by calculating the blue-stained areas in 10 random different views.

### Prussian blue staining

Histological sections were stained with Prussian blue (G1422, Solarbio, China) to detect iron deposition, according to the manufacturer’s instructions. Representative images were captured under a microscope (DP73, OLYMPUS, Japan; magnification, 200×). Integrated optical density (IOD) of ferrous iron deposit areas was measured using Image-Pro Plus 6.0 and average densities calculated as IOD/area.

### Immunohistochemistry (IHC)

Tissue sections were deparaffinized, rehydrated, and subjected to antigen retrieval, then stained with primary antibodies as flows: neutrophil gelatinase-associated lipocalin (NGAL, 1:200, Ab216462, Abcam, UK), 4-HNE (1:200, MAB3249-SP, NOVUS, USA), according to the IHC kit instructions (DAB-0031, KIT-9710, MXB, China). Samples were dehydrated, cleared, and sealed and representative images captured by microscopy (DP73, OLYMPUS, Japan; magnification, 400×). NGAL and 4-HNE IOD values were calculated as for Prussian blue staining.

### Immunofluorescence (IF)

Paraffin-embedded renal tissue samples were sliced into 5-μm-thick sections, which were deparaffinized and rehydrated in a graded ethanol series. Antigen retrieval was achieved by boiling sections in antigen recovery buffer (10 min), followed by incubation in goat serum (15 min). Sections were then incubated with primary antibodies against GPX4 (1:200, DF6701, Affinity, China) and SLC7A11 (1:200, NB300-317SS, NOVUS, USA) (4 °C, overnight), rinsed three times, and immersed in Cy3-conjuated goat anti-rabbit IgG (1:200, A0516, Beyotime, China) (60 min, room temperature, in darkness). Cell nuclei were stained with DAPI (D106471-5 mg, Aladdin, China) and images captured using a fluorescence microscope (magnification, 400×).

### TUNEL assay

Dewaxed and dehydrated paraffin sections were washed with distilled water and processed for TUNEL staining according to the manufacturer’s instructions (C1088, Beyotime, China). After sealing, sections were observed under a fluorescent microscope (magnification, 400×) and the numbers of green cells in eight random views counted.

### Real-time PCR (RT-qPCR)

Total RNA was extracted from frozen renal tissue using TRIPure (RP1001, BioTeke Corporation, China), according to the manufacturer’s protocol. Isolated RNA samples were reverse transcribed into cDNA using super M-MLV reverse transcriptase (PR6502, BioTeke Corporation, China), then subjected to RT-qPCR using SYBR Mix and corresponding primers on a PCR cycler (Exicycler 96, BIONEER, Korea). The internal reference was *β-actin*, which was used for data normalization in quantitative analysis. Primers (Table 1) were synthesized by GenScript Biotechnology (Nanjing, China).

**Table 1:** Sequences of primer pairs used for gene amplification in this study.

Target gene	Forward primer (5′–3′)	Reverse primer (5′–3′)
*GPX4*	GAGGCAGGAGCCAGGAAGT	ACAGTGGGTGGGCATCGTC
*NGAL*	GATGAACTGAAGGAGCGATTC	TCGGTGGGAACAGAGAAAAC
*β-actin*	CACTGTGCCCATCTACGAGG	TAATGTCACGCACGATTTCC

### Western blot (WB) assays

Total proteins were extracted from renal tissues using a kit (BC3710, Solarbio, China), and quantified by BCA assay kit (P0010, Beyotime, China). Equal amounts of protein samples were loaded onto 6%, 8%, 10%, 12%, and 15% SDS-polyacrylamide gels and subjected to electrophoresis. Proteins were then transferred onto PVDF membranes, blocked with 5% dried skimmed milk (room temperature, 1 h), and incubated with primary antibodies against: GPX4 (1:4000, Ab125066, Abcam, UK), ACSL4 (1:8000, Ab155282, Abcam, UK), cleaved-caspase3 (1:500, WL02117, Wanleibio, China), B-cell lymphoma-2 (Bcl-2, 1:500, WL01556, Wanleibio, China), Bcl-2-associated X (Bax, 1:1500, T40051, Abmart, China), heme oxygenase-1 (HO-1, 1:750, WL02400, Wanleibio, China), divalent metal transporter 1 (DMT1, 1:1000, Ps-3577, Abmart, China), transferrin receptor (TfR, 1:1250, T56618, Abmart, China), ferritin heavy chain (FtH, 1:1250, T55648, Abmart, China), ferritin light chain (FtL, 1:2000, T56955, Abmart, China), nuclear receptor coactivator 4 (NCOA4, 1:500, A5695, ABclonal, China), ferroportin (FPN, 1:1000, TD13561, Abmart, China), α-smooth muscle actin (α-SMA, 1:3000, 14395-1-AP, proteintech, China), collagen type I (COL I, 1:1000, Ab270993, Abcam, UK), transforming growth factor β1 (TGF-β1, 1:1000, Ab215715, Abcam, UK), Smad3 (1:2000, Ab40854, Abcam, UK), p-Smad3 (1:2000, Ab52903, Abcam, UK) and GAPDH (1:25000, 60004-1-Ig, proteintech, China) (4 °C, overnight). After washing, blots were incubated with HRP-conjugated secondary antibodies (1:25000, E030120-01, E030110-01, Earthox, USA) at room temperature for 1 h. Gray values of specific bands were measured using ImageJ software; each band was measured in triplicate and experiments were repeated three times.

### Statistical analysis

Data are presented as mean ± SD. Normality and homogeneity of variance were verified, and one-way ANOVA analysis was performed using GraphPad Prism 8.0 (GraphPad Software, San Diego, CA). Tukey’s test was used to determine the significance of differences among groups. *P* < 0.05 were considered significant.

## Supplementary information

Original Data File

Author Contribution Statement

## Data Availability

The data used to support the findings of this study are available from the corresponding author upon request.
