# Supplementary material for: Ferroptosis, a new target for treatment of renal injury and fibrosis in a 5/6 nephrectomy-induced CKD rat model
Source: Cell Death Discov. 2022 Mar 22;8:127. doi: 10.1038/s41420-022-00931-8 (PMC8941123; doi:10.1038/s41420-022-00931-8)

GPX4

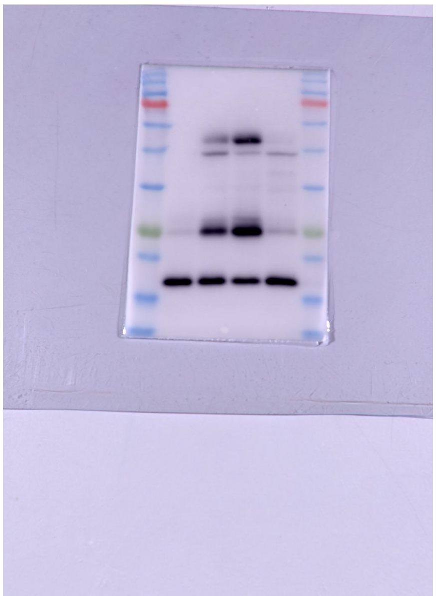

ACSL4

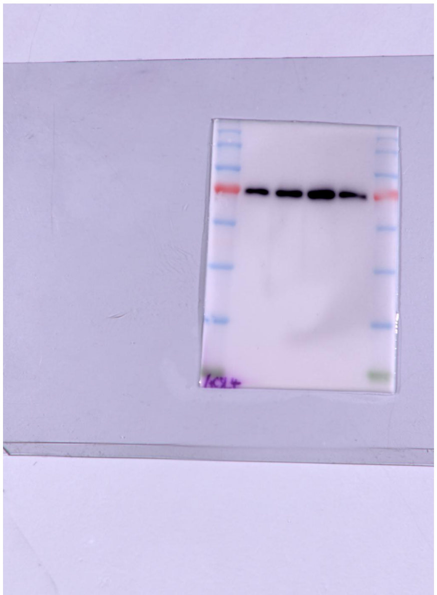

GAP

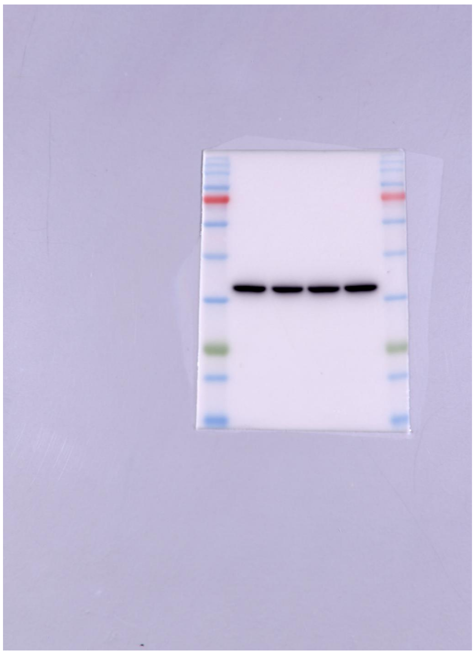

Cleaved-caspase3

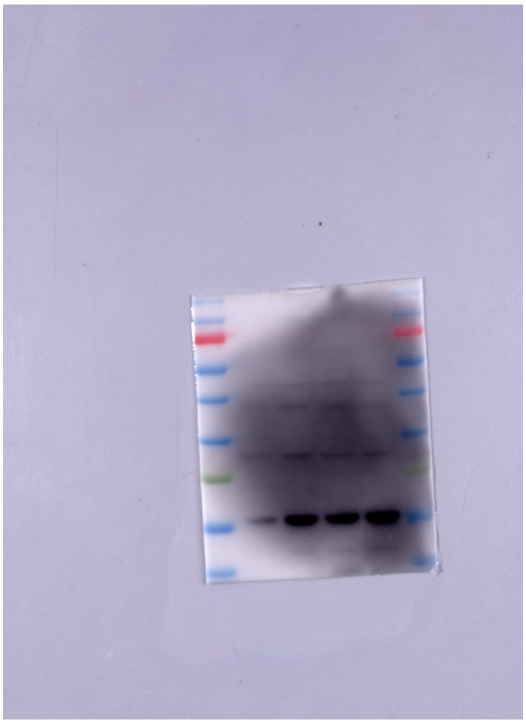

Bcl-2

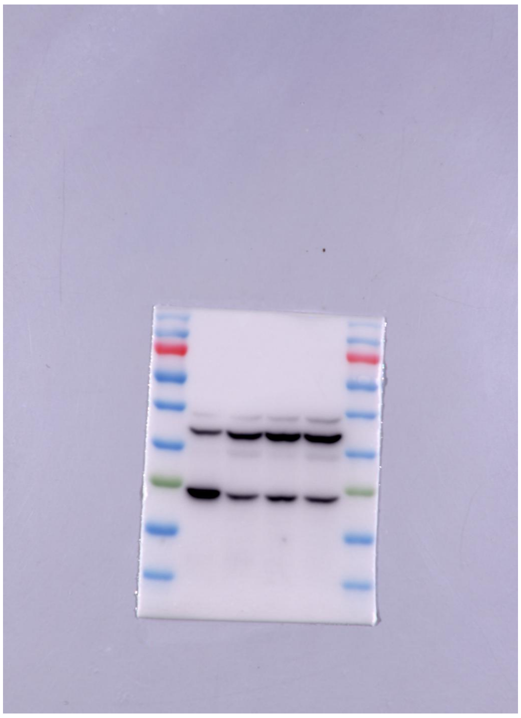

Bax

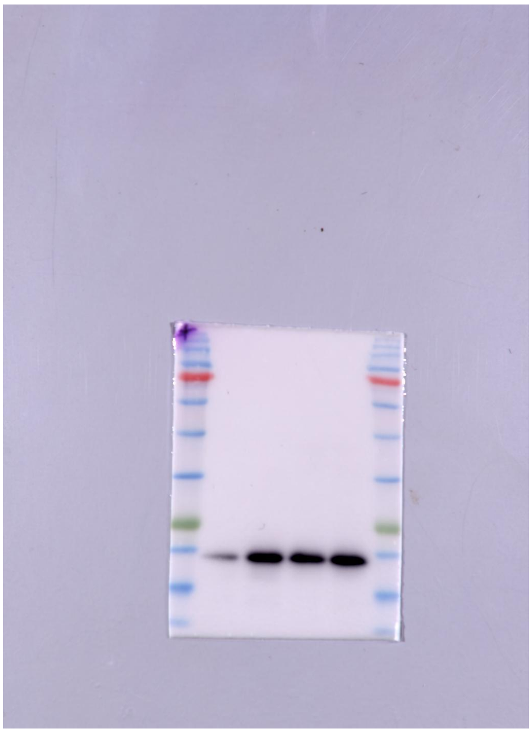

GAP

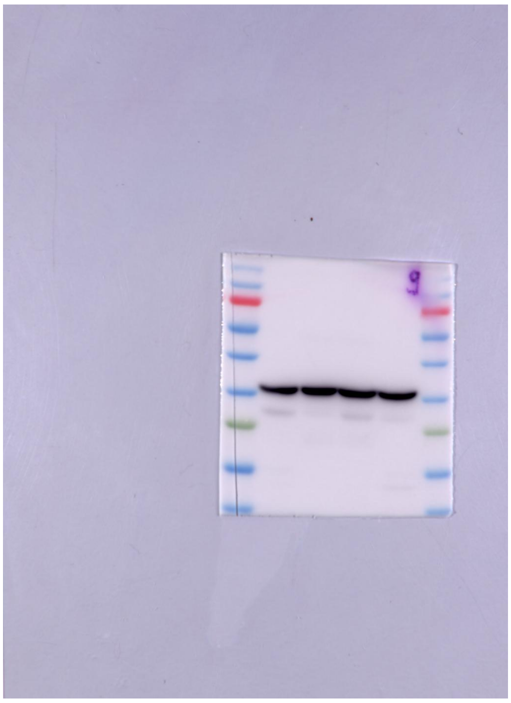

HO-1

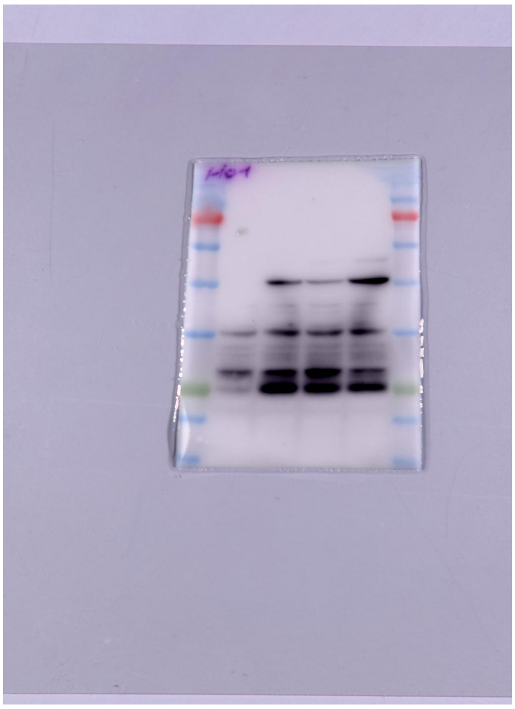

DMT1

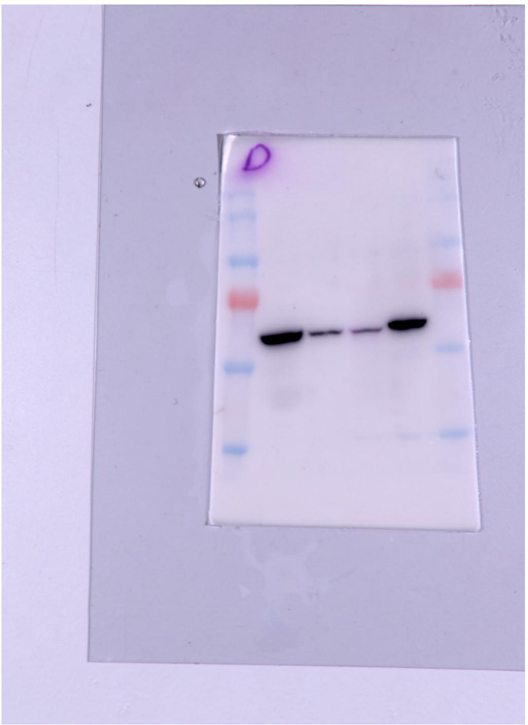

TfR

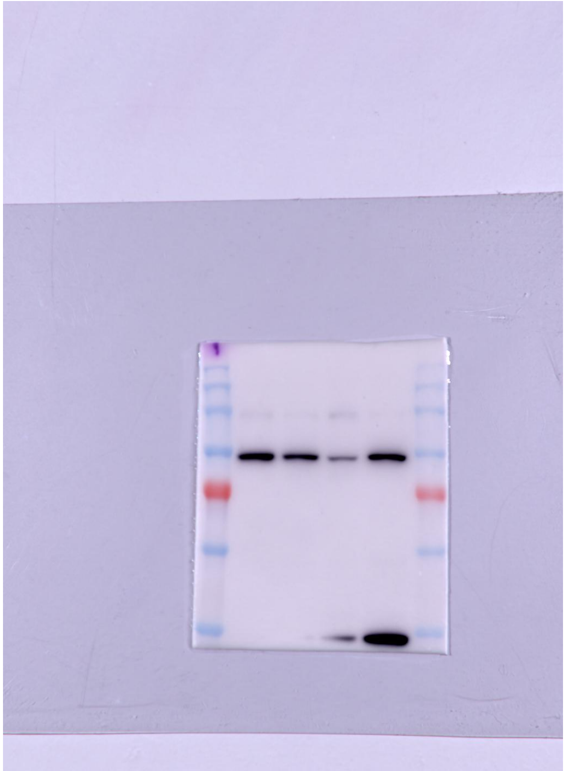

FtH

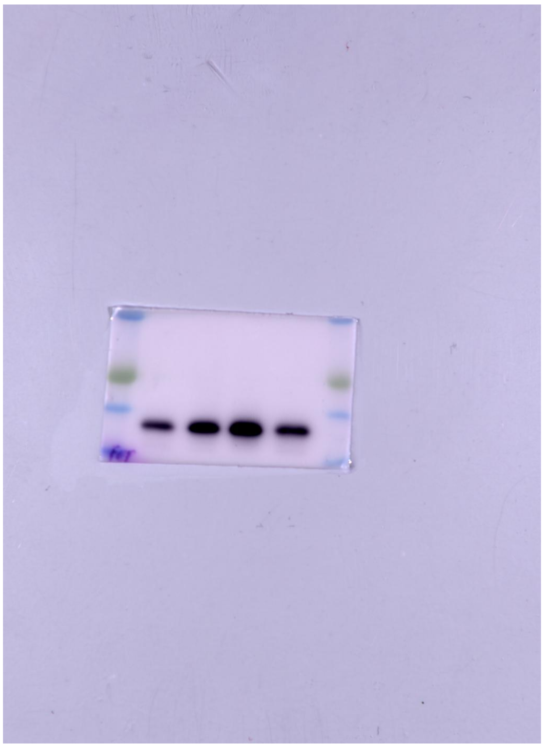

FtL

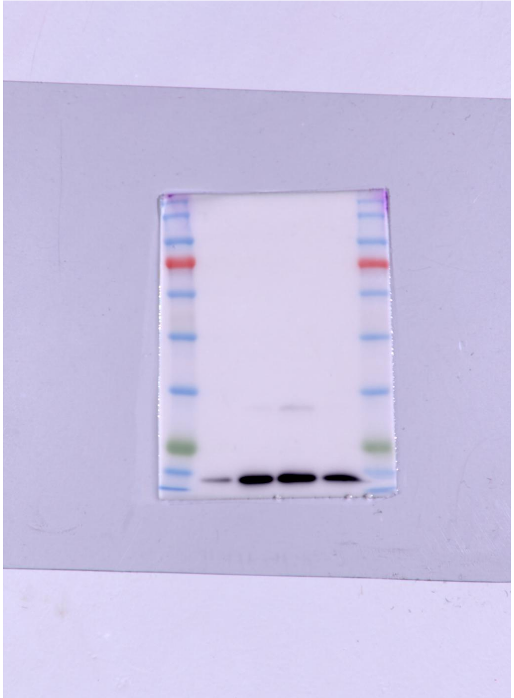

NCOA4

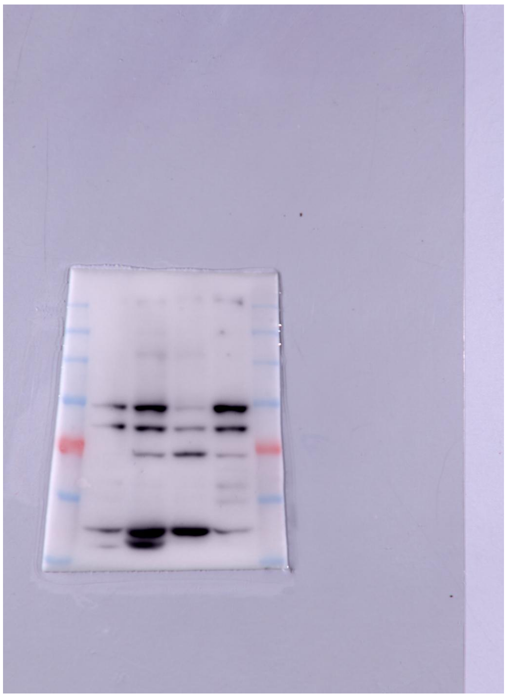

FPN

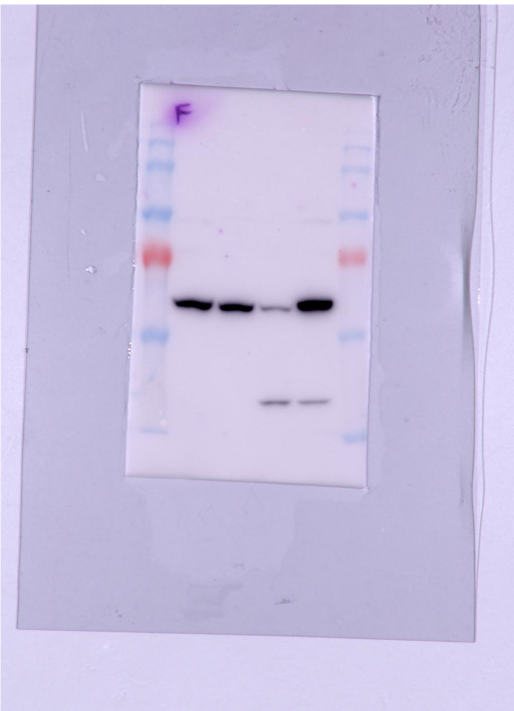

GAP

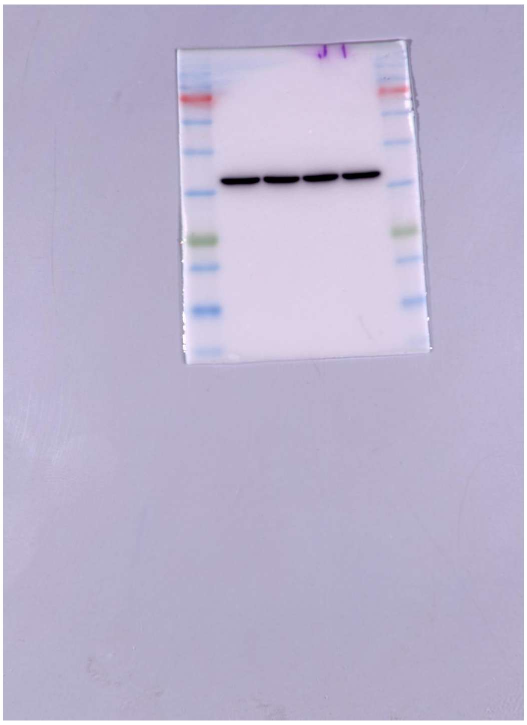

α -SMA

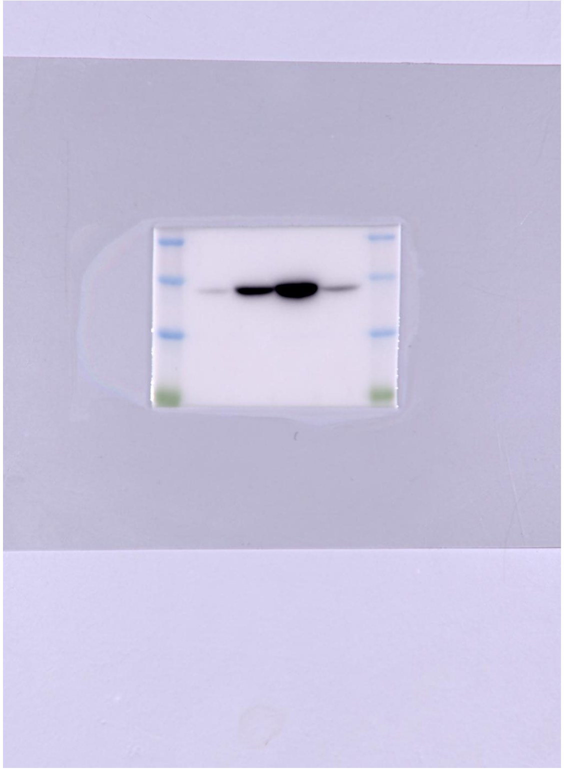

COL Ⅰ

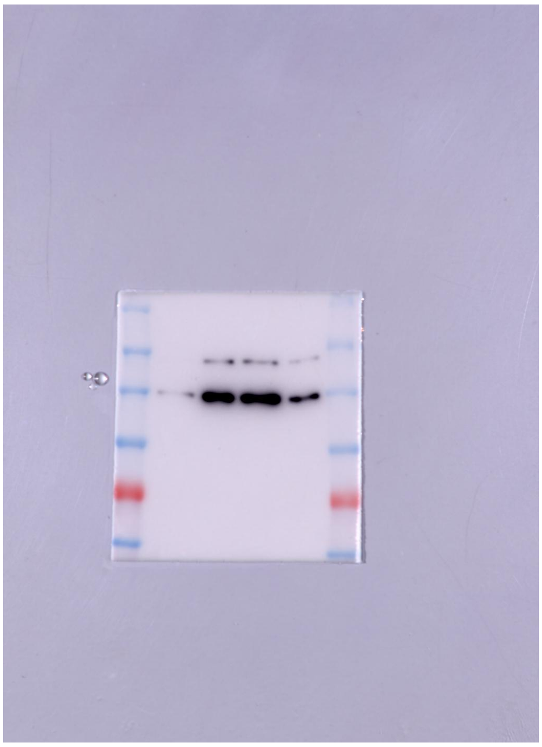

GAP

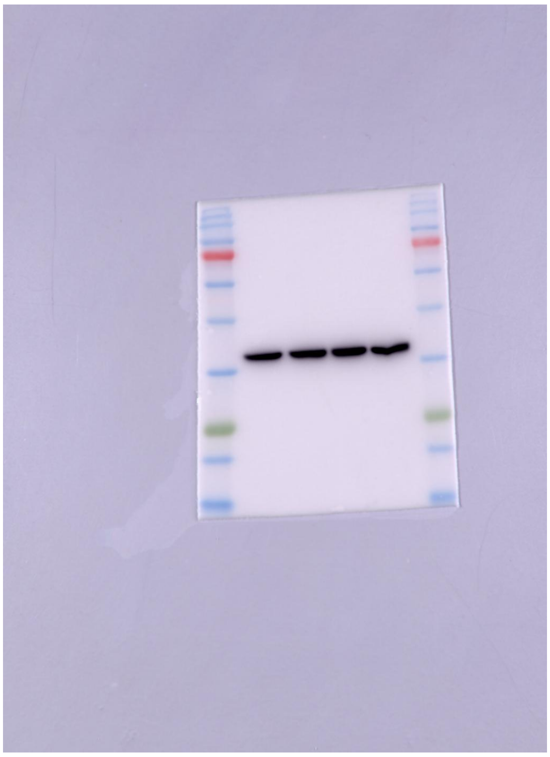

TGF- β 1

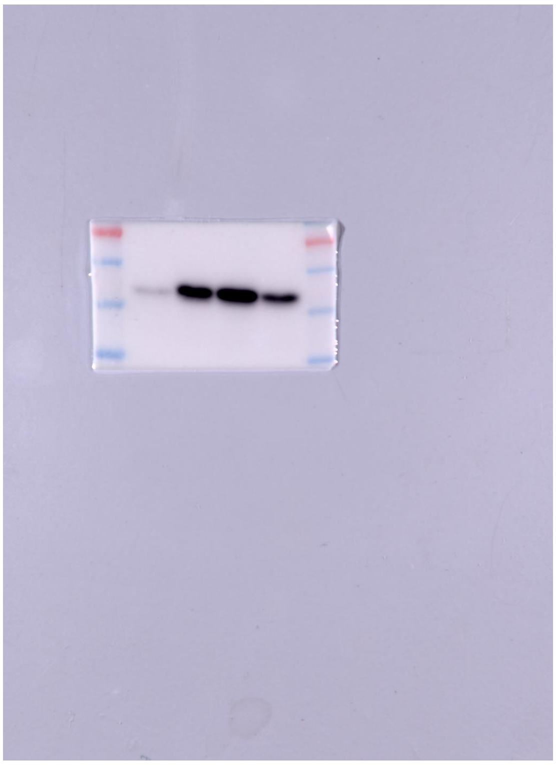

Smad3

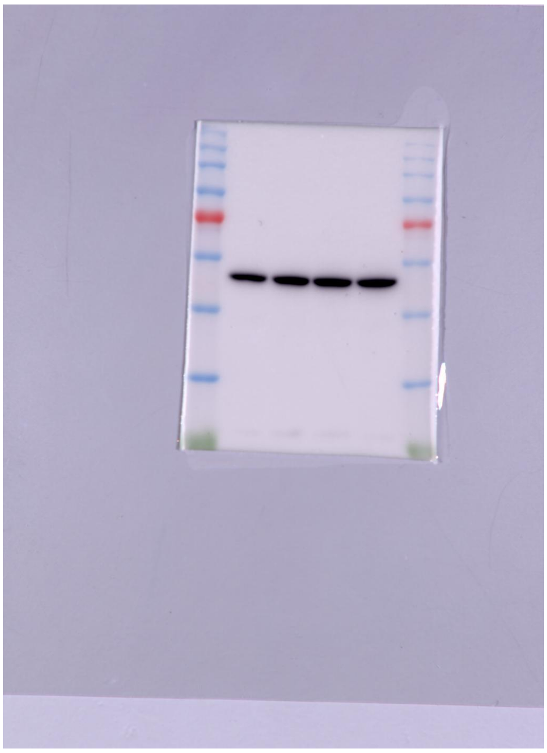

p-Smad3

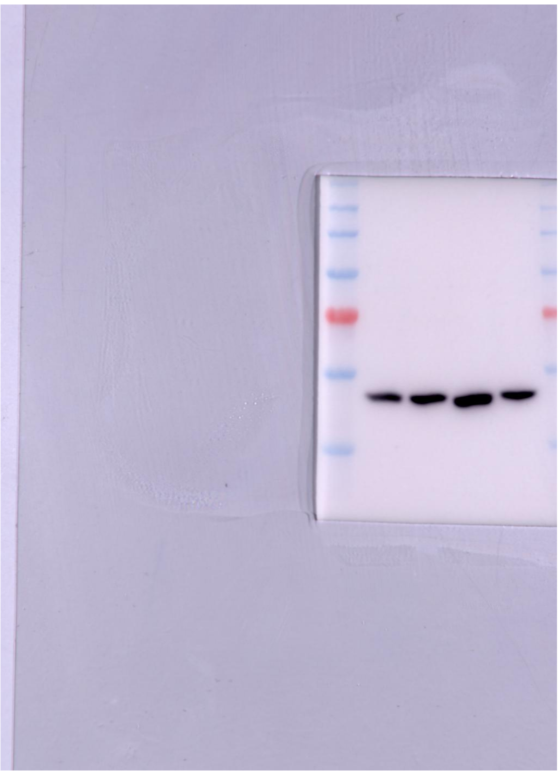

GAP

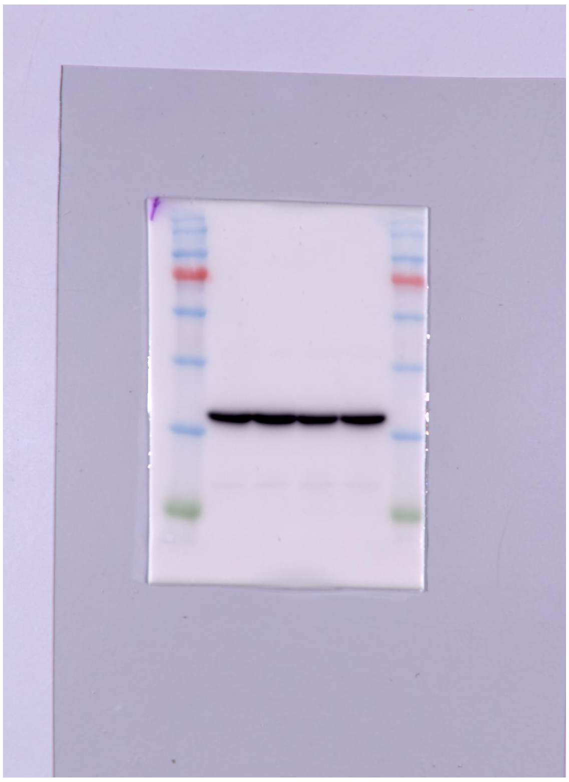

Supplement: Supplementary file 1 — Original Data File [file 41420_2022_931_MOESM1_ESM.pdf]
