# Supplementary material for: Ferroptosis, a new target for treatment of renal injury and fibrosis in a 5/6 nephrectomy-induced CKD rat model
Source: Cell Death Discov. 2022 Mar 22;8:127. doi: 10.1038/s41420-022-00931-8 (PMC8941123; doi:10.1038/s41420-022-00931-8)
Supplement: Supplementary file 2 — Author Contribution Statement [file 41420_2022_931_MOESM2_ESM.docx]

**Author Contribution Statement**

Jingyu Wang. and Li Sun. performed study concept and design; Jingyu Wang., Yaqing Wang., Xin Huang., Wenjing Fu and Li Sun. performed development of methodology and writing, review and revision of the paper; Yi Liu., Xintian Cai., Lei Wang., Lihua Qiu., and Junying Li. provided acquisition, analysis and interpretation of data, and statistical analysis; Li Sun. provided financial support. All authors read and approved the final paper.
